# Generalized Drug Eruption Secondary to Ticagrelor: A Case Report and Review of the Literature

**DOI:** 10.7759/cureus.34800

**Published:** 2023-02-09

**Authors:** Raziye E Akdogan, Jennifer Chen, Alberto Varon, Elnara Muradova, Joseph Fusco

**Affiliations:** 1 Internal Medicine, University of Connecticut School of Medicine, Farmington, USA; 2 Dermatology, University of Connecticut School of Medicine, Farmington, USA; 3 Internal Medicine, St. Francis Hospital, Hartford, USA

**Keywords:** thienopyridines, antiplatelets, hypersensitivity rash, drug-eruption, ticagrelor hypersensitivity

## Abstract

Currently, guidelines recommend ticagrelor over clopidogrel as part of dual antiplatelet therapy with aspirin in treating individuals with acute coronary syndrome. As there is an increased usage of ticagrelor, it is important to keep in mind uncommon adverse events, including hypersensitivity skin reactions. To date, only a few studies have been published regarding ticagrelor-induced skin eruptions. Additionally, there is no consensus on antiplatelet therapy management after a hypersensitivity reaction to antiplatelet agents. Hereinafter, we describe a case of an 81-year-old female who presents with a diffuse erythematous hypersensitivity eruption, including palms and soles, secondary to ticagrelor use. Ticagrelor transitioned to clopidogrel, and the patient was started on steroid taper with an antihistamine. The patient’s rash progressively improved after the treatment. Our case demonstrates a rare adverse effect of ticagrelor, which needs prompt diagnosis and switching to one of the thienopyridines to prevent thrombosis.

## Introduction

Ticagrelor is a relatively new antiplatelet medication introduced in 2011, which has increased in practice after many guidelines recommended using ticagrelor over clopidogrel for acute coronary syndromes [1]. This recommendation was communicated after the Platelet Inhibition and Patient Outcomes (PLATO) study [2], which demonstrated that ticagrelor had a 16% relative risk reduction in major adverse coronary events and a 22% relative risk reduction in all-cause death compared with those treated with clopidogrel. Ticagrelor’s common side effects include dyspnea, bleeding, and bradyarrhythmias while skin-related adverse events are limited. Upon a literature search, we are only able to identify six cases describing a hypersensitivity skin reaction after the initiation of ticagrelor. In this case report, we describe an 81-year-old female with a new diffuse erythematous rash involving the palms and soles secondary to ticagrelor use.

## Case presentation

**Figure 1:**
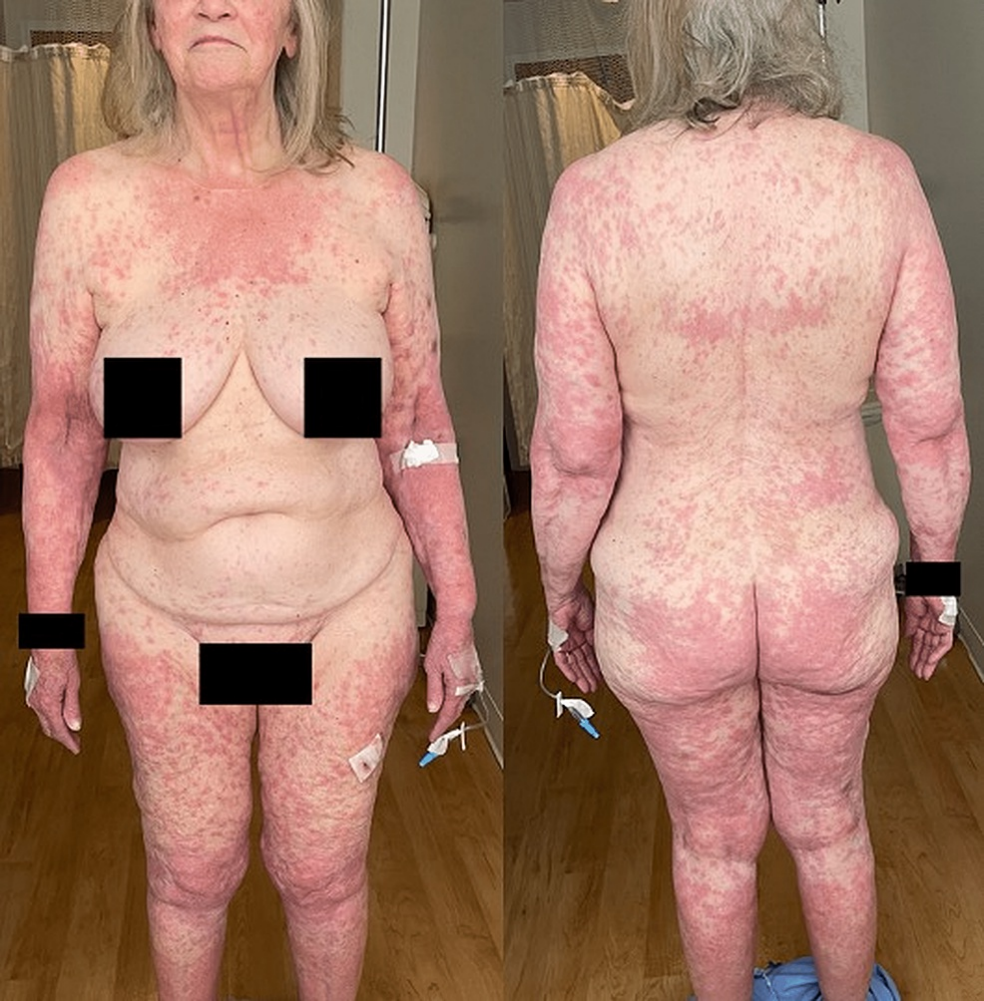
Anterior and posterior cutaneous findings Generalized erythematous non-blanching coalescing macules, papules, and patches, involving the bilateral palms and soles.

**Figure 2:**
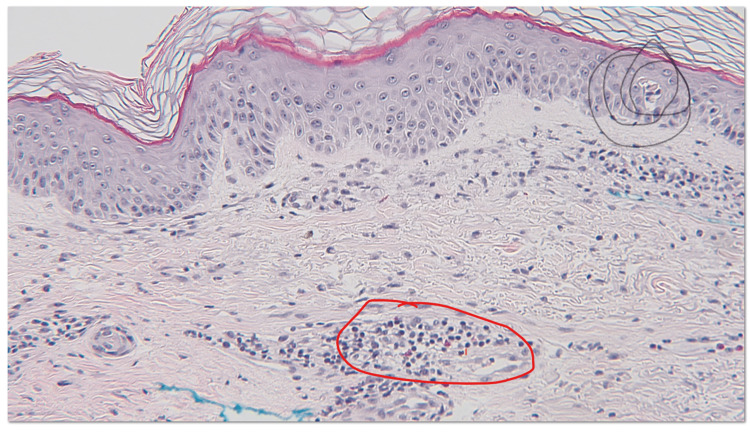
H&E skin biopsy from the left lateral thigh Perivascular lymphocytic spongiotic dermatitis with scattered eosinophils (red circle) and rare necrotic keratinocytes (black spiral), consistent with a drug eruption.

An 81-year-old Caucasian female with a past medical history of coronary artery disease, hypertension, hypothyroidism, osteoarthritis, gastroesophageal reflux disease, and recurrent urinary tract infections presents to the emergency department with a one-day history of pruritic generalized erythematous tender eruption involving bilateral acral surfaces. Ten days prior to the onset of the rash, the patient was admitted for non-ST elevation myocardial infarction and had a drug-eluting stent placed in the circumflex artery. She was then started on ticagrelor. The patient denied any other new medications, over-the-counter supplements, and new detergents or soaps. Additionally, the patient denied any fever, arthralgia, blisters, dyspnea, facial edema, difficulty swallowing, or dysuria. Her vitals showed a systolic blood pressure in the 70s mmHg, which improved after fluid resuscitation. She was afebrile with a heart rate in the 60s. For laboratory work-up, she had an increased WBC count to 18.1 (K/µL) with a left shift without eosinophilia. Her chemistries showed creatinine at 1.2 mg/dl, sodium at 132 meq/ L, potassium at 3.1 meq/L, estimated glomerular filtration rate (eGFR) at 43.1 ml/min, and a sedimentation rate of 45 mm/h. On physical examination, erythematous non-blanching coalescing macules, papules, and patches were localized on the face, trunk, and extremities, as well as bilateral palms and soles. There was no mucosal involvement (Figure 1). A skin biopsy of the left lateral thigh demonstrated perivascular lymphocytic spongiotic dermatitis with scattered eosinophils and rare necrotic keratinocytes, consistent with a drug eruption (Figure 2).

For management, ticagrelor was switched to clopidogrel, and she was started on steroids (methylprednisolone IV followed by a tapering dose of oral prednisone) and an antihistamine (IV diphenhydramine). Over a three-day hospital course, the patient's eruption and pruritus gradually improved, starting at the palms and soles.

## Discussion

This is the first case of generalized drug-induced eruption involving bilateral palms and soles, secondary to ticagrelor hypersensitivity. To the best of our knowledge, there were only six other case reports published regarding ticagrelor hypersensitivity skin reactions. Two of the case reports demonstrated maculopapular rash that mainly involved only the central trunk as opposed to our case, which involved the entire body, including palms and soles [3,4]. The case reported by Quinn et al. demonstrated very similar skin biopsy findings showing perivascular lymphocytic infiltrates compared to our patient but did not comment on necrotic keratinocytes as seen in our biopsy [3]. The four other reports showed different dermatological reactions with one case of exanthematous pustulosis [5], one case of eccrine hidradenitis [6], one case of a bullous fixed drug eruption [7], and a case of Sweet syndrome [8]. These cases are described in Table 1.

**Table 1:** Case reports of ticagrelor-induced hypersensitivity skin reactions

Age	Gender	Cutaneous Findings	Biopsy Findings	Treatment	Reference
65	M	Pruritic exanthematous rash	Perivascular lymphocytic infiltrate	Discontinuation of the offending drug	Quinn et al. 2014 [3]
68	M	Pruritic and exanthematous eruptions	None provided	Switched to warfarin due to clopidogrel resistance	Dai et al. 2017 [4]
65	F	Generalized erythroderma with scattered sloughing, with accentuation on the abdomen and thighs	Acute generalized exanthematous pustulosis	Did not mention	Maybrook et al. 2015 [5]
73	M	Multiple painful hemorrhagic bullae on the palms of both hands	Neutrophilic infiltrate consistent with Sweet Syndrome	Switched to clopidogrel	Ikram and Kandasamy 2017 [8]
66	M	Painful erythematous papulonodules on both palms	Revealed dermal edema and infiltration of eccrine gland coils and ducts by a dense neutrophilic and eosinophilic infiltrate	Switched to clopidogrel	Bishnoi et al. 2019 [6]
44	M	Translucent bullous lesions with surrounding erythema on the distal flexor aspect of the right forearm	None performed	Switched to clopidogrel and rivaroxaban	Kawall et al. 2021 [7]
81	F	Erythematous maculopapular rash involving her palms and soles	Perivascular lymphocytic dermatitis, spongiotic type, with scattered eosinophils and rare necrotic keratinocytes	Switched to clopidogrel	Our case

Symptomatic management with antihistamines and steroids and discontinuation of the offending agent are the mainstays of the treatment. In addition, antithrombotic therapy should continue to prevent further ischemic events by switching to another class of antiplatelet agents. Among these seven cases reported, including ours, four were successfully transitioned to clopidogrel without recurrence of skin lesions. Notably, one case was ultimately changed to warfarin due to recurrent thrombosis after switching to clopidogrel [4]. Although clopidogrel and ticagrelor are both antiplatelets that are P2Y12 adenosine diphosphate (ADP) receptor blockers, they are structurally different. P2Y12 ADP receptor blockers are split into two categories based on their chemical structures: thienopyridines and cyclopentyl-triazole-pyrimidines (CTP). Thienopyridines irreversibly inhibit platelet activation by binding ADP receptors and include clopidogrel, prasugrel, and ticlopidine. CTP reversibly binds ADP receptors, that is, ticagrelor and cangrelor [9]. Therefore, cross-reactive hypersensitivity is less likely to occur when changing from a CTP to thienopyridines, as observed in the analyzed cases above.

Identified skin adverse events from ticagrelor appear to be at a much lower incidence compared to clopidogrel, as up to 6% of patients will develop a clopidogrel-induced rash [10]. Although these dermatological events appear to be uncommon in patients receiving ticagrelor, it may be due to its more recent introduction, and we might expect to see an increase in these events as ticagrelor utilization continues to rise [1].

## Conclusions

This case highlights the importance of considering ticagrelor as a potential cause of a pruritic generalized erythematous hypersensitivity rash of the whole body, including palms and soles. Prompt identification allows for a quick replacement of the CTP offending agent with one of the thienopyridines. Cross-reactive hypersensitivity is rare given CTP, and thienopyridines are structurally different. This will allow for the resolution of the hypersensitivity as well as the continuation of antithrombotic therapy to prevent a catastrophic ischemic event.

## References

[1] Henriksson R, Ulvenstam A, Söderström L, Mooe T (2019). Increase in ticagrelor use over time is associated with lower rates of ischemic stroke following myocardial infarction. BMC Cardiovasc Disord.

[2] Turgeon RD, Koshman SL, Youngson E, Har B, Wilton SB, James MT, Graham MM (2020). Association of ticagrelor vs clopidogrel with major adverse coronary events in patients with acute coronary syndrome undergoing percutaneous coronary intervention. JAMA Intern Med.

[3] Quinn KL, Connelly KA (2014). First report of hypersensitivity to ticagrelor. Can J Cardiol.

[4] Dai J, Lyu S, Ge C (2017). Hypersensitivity to ticagrelor and low response to clopidogrel: a case report. Asia Pac Allergy.

[5] Maybrook RJ, Fischer R, Deibert B (2015). Ticagrelor-induced acute generalized exanthematous pustulosis. Int J Cardiol.

[6] Bishnoi A, Daroach M, Aggarwal D, Radotra BD, Panda P, Parsad D (2019). Ticagrelor induced neutrophilic eccrine hidradenitis: a unique adverse effect of a new antiplatelet drug. Postgrad Med J.

[7] Kawall T, Seecheran R, Seecheran V, Persad S, Seecheran NA (2021). Suspected ticagrelor-induced bullous fixed drug eruption. Cureus.

[8] Ikram S, Veerappan Kandasamy V (2017). Ticagrelor-induced Sweet syndrome: an unusual dermatologic complication after percutaneous coronary intervention. Cardiovasc Interv Ther.

[9] Wallentin L, Becker RC, Budaj A (2009). Ticagrelor versus clopidogrel in patients with acute coronary syndromes. N Engl J Med.

[10] Campbell KL, Cohn JR, Fischman DL, Walinsky P, Mallya R, Jaffrani W, Savage MP (2011). Management of clopidogrel hypersensitivity without drug interruption. Am J Cardiol.

